# Editorial: Women in plant pathogen interactions: 2022

**DOI:** 10.3389/fpls.2023.1249821

**Published:** 2023-07-25

**Authors:** Špela Baebler, Anna Coll, Giulia Malacarne

**Affiliations:** ^1^ Department of Biotechnology and Systems Biology, National Institute of Biology, Ljubljana, Slovenia; ^2^ Research and Innovation Center, Fondazione Edmund Mach, San Michele all’Adige, Italy

**Keywords:** plant pathogen, bacteria, fungi, viruses, beneficial microbes, plant-pathogen interaction, plant protection, physical barrier

Plants are continuously exposed to different pathogens and pests which can lead to devastating effects on agricultural production. A better understanding of plant defence response against pathogens is crucial to provide means for novel crop breeding strategies and environmentally friendly plant protection and disease management approaches. In this Research Topic, we aimed to highlight the diversity of research performed across the entire breadth of the plant-pathogen interactions field. It thus includes the most recent scientific advances in understanding the molecular, cellular, and biochemical mechanisms of plant response to bacteria, fungi, and viruses with applications to compelling problems. In the Research Topic, six research papers (one about plant-bacterial interaction, three about plant-fungal interactions, and two about plant-virus interaction) and a review were published and are outlined below.

During their life, plants interact with many pathogenic microorganisms which are also controlled by physical barriers. The second physical barrier they encounter after the cuticle is the cell wall (Choi et al., 2021). Therefore, pathogens require active plant cell wall-modifying proteins to be successful in their interaction with the plant host (Bellincampi et al., 2014). At the same time, altered cell wall properties by the host might be responsible for pathogen resistance or susceptibility outcomes (Hou et al., 2019). In this context, Narváez-Barragán et al. reviewed the plant immune response activated by cell wall surveillance mechanisms. In particular, they focused on the mechanism related to cell wall-derived fragments which, sensed by the host, trigger defence responses and reinforcement of the cell wall, in which expansin proteins might also be involved.

Plant pathogenic bacteria *Ralstonia solanacearum* is the causative agent of bacterial wilt of potato (brown-rot) and other vegetable crops (Janse, 1996). As a consequence of climate change, recycling of irrigation water has become an alternative for potato production, in which irrigation is essential to ensure a high tuber yield (Alva, 2008). The only limitation is that contaminated irrigation water contributes significantly to the spread of the pathogen. However, the exact biological threshold to cause the infection is still unknown (EFSA et al., 2019). In this sense, dose-response models are essential in risk assessments. Therefore, Eisfeld et al. aimed to develop a dose-response model for *R. solanacearum* to be adopted in management strategies to provide safe irrigation water, reducing or eliminating the risk of bacterial wilt infection.

Fungi and oomycetes pathogens are the most widespread crop pests and pathogens (Bebber et al., 2014) posing a significant impact on crop yield and productivity. Among them, *Colletotrichum truncatum* is the main causing agent of anthracnose disease in soybean and can lead to substantial yield losses. Boufleur et al. investigated the transcriptome response of two soybean cultivars presenting different degrees of susceptibility to two different *C. truncatum* strains. The study showed that the response of the more resistant phenotypes resulted in a set of common differentially expressed genes involved in different immune response pathways, which might help to elucidate key soybean defence pathways against *C. truncatum*.

The key role of phytohormones in plant immunity is largely known, in particular, salicylic acid (SA) and jasmonic acid (JA) have been shown to contribute to plant defence response against different pathogens. Cytokinins (CKs) can have an impact on SA- and JA-mediated defence response (Argueso et al., 2012; Zhang et al., 2022). However, the effect of the triple hormonal treatment was not reported until now. Still, with the aim of uncovering molecular mechanisms of plant defence against fungi, Falconieri et al. demonstrated the involvement of SA/JA/CK treatment and the role of type-B response regulator 11, implicated in CK-mediated responses, on Arabidopsis fitness and resistance against the fungal necrotrophic pathogen *Botrytis cinerea*.

Interactions between plants and microorganisms are highly diverse and can also be beneficial. The latter include endophytes, a group of bacteria and fungi inhabiting various internal plant tissues without causing disease symptoms. An example of it is an *Acrophialophora jodhpurensis* fungal isolate that has been isolated and characterised by Daroodi et al.. The authors proved the antagonistic activity of the endophytic fungus against *Alternaria alternata*, the causal agent of early blight in tomatoes, and showed that the endophyte also enhances tomato growth parameters. These results open the door for the potential use of *A. jodhpurensis* as a biological fertiliser and biocontrol agent.

Plant viruses are obligate parasites and must manipulate, reprogram and redirect the host’s cellular machinery to replicate inside the plant cell. Plant response to geminivirus invasion is mediated by RNA-directed DNA methylation to suppress the minichromosomes, formed by geminivirus replication (Zarreen and Chakraborty, 2020). Mulaudzi et al. analysed methylation differences between tomato curly stunt virus (ToCSV) a susceptible and a tolerant tomato genotype. The extent of methylation of viral cytosine residues was more pronounced in the tolerant, compared to susceptible genotype, providing cues for understanding the mechanism of tolerance in this less investigated pathosystem.

Plant viruses are usually part of a broader community that includes other plant-associated microbes and pests. For example, insect vectors of plant viruses can facilitate the transmission of viruses by modulating plant chemistry to attract the vector (Xavier and Whitfield, 2023). Kwon et al. have shown that aphids (*Myzus persicae and Aphis glycines*) were more attracted to Cucumber mosaic virus (CMV) - infected pepper plants than to healthy plants and confirmed that the phenomenon can be attributed to increased production of ethylene in CMV-infected plants, as confirmed by transcriptome and volatile analysis.

This Research Topic sheds light on molecular mechanisms underlying the plant defence response and provides new applicative results in the plant protection field. Moreover, an additional aim of the Research Topic was to encourage women, minorities, and early-career researchers to contribute to it. It is thus a noteworthy achievement that women are represented above average: out of seven published papers, there were seven and three with the first and last female authors, respectively, and five papers with predominantly female authors. Last but not least, the editors would like to thank all contributors to this Research Topic.

## Author contributions

All authors listed have made a substantial, direct, and intellectual contribution to the work and approved it for publication.
